# Variations in mitochondrial cytochrome *b* region among Ethiopian indigenous cattle populations assert *Bos taurus* maternal origin and historical dynamics

**DOI:** 10.5713/ajas.17.0596

**Published:** 2018-04-11

**Authors:** Getinet Mekuriaw Tarekegn, Xiao-yang Ji, Xue Bai, Bin Liu, Wenguang Zhang, Josephine Birungi, Appolinaire Djikeng, Kassahun Tesfaye

**Affiliations:** 1 Department of Animal Breeding and Genetics, Swedish University of Agricultural Sciences, Uppsala P.O. Box 7070, Sweden; 2 Department of Animal Production and Technology, Bahir Dar University, Bahir Dar P.O. Box 79, Ethiopia; 3 College of Animal Science, Inner Mongolia Agricultural University, Hohhot 010018, China; 4 Nei Mongol BioNew Technology Co. Ltd, Hohhot 010020, China; 5 Biosciences Eastern and Central Africa (BecA) Hub - International Livestock Research Institute, PO Box 30709, Nairobi 00100, Kenya; 6 Centre for Tropical Livestock Genetics and Health, The University of Edinburgh, Easter Bush, Midlothian, EH25 9RG, Scotland, UK; 7 Department of Microbial Cellular and Molecular Biology, Addis Ababa University, Addis Ababa P.O. Box, 1176, Ethiopia

**Keywords:** Cytochrome *b*, Ethiopian Cattle, Haplotype Diversity, Population Expansion

## Abstract

**Objective:**

This study was carried out to assess the haplotype diversity and population dynamics in cattle populations of Ethiopia.

**Methods:**

We sequenced the complete mitochondrial cytochrome *b* gene of 76 animals from five indigenous and one Holstein Friesian×Barka cross bred cattle populations.

**Results:**

In the sequence analysis, 18 haplotypes were generated from 18 segregating sites and the average haplotype and nucleotide diversities were 0.7540±0.043 and 0.0010±0.000, respectively. The population differentiation analysis shows a weak population structure (4.55%) among the populations studied. Majority of the variation (95.45%) is observed by within populations. The overall average pair-wise distance (*F*_ST_) was 0.049539 with the highest (*F*_ST_ = 0.1245) and the lowest (*F*_ST_ = 0.011) *F*_ST_ distances observed between Boran and Abigar, and Sheko and Abigar from the indigenous cattle, respectively. The phylogenetic network analysis revealed that all the haplotypes detected clustered together with the *Bos taurus* cattle and converged to a haplogroup. No haplotype in Ethiopian cattle was observed clustered with the reference *Bos indicus* group. The mismatch distribution analysis indicates a single population expansion event among the cattle populations.

**Conclusion:**

Overall, high haplotype variability was observed among Ethiopian cattle populations and they share a common ancestor with *Bos taurus*.

## INTRODUCTION

Cattle are believed to have originated from *Bos primigenius* in the southwest Asia (*Bos taurus*) between 8,000–10,000 YA and south Asia (*Bos indicus*) [1,2], and spread throughout the old world following the human trade and migration [3]. The Horn and North of Africa are considered to be the ancient gateways for the dispersal of domesticates into the African continent [2,4]. The *Bos taurus* group arrived in these regions, in Ethiopia in particular, around 7,000 years BC [5]. Those regions are the cradle of both Near-East *Bos taurus*, and Arabian and Indian *Bos indicus* cattle migration corridor and sometime considered the secondary hybridization zone [6,7]. The *Bos indicus* cattle descended from the putative cattle domestication center in the northern part of the Indian subcontinent, the Indus Valley [8] and arrived to East Africa between 2,000–3,000 BC [9]. Conversely, the world-wide genome-wide analysis of ancestry, divergence and admixture revealed that African taurine cattle were first domesticated in the Middle East and later hybridized with African aurochs [10].

Being the major entry point of cattle to Africa, the genetic landscape of the current cattle populations in Ethiopia could have been shaped by several introductions of zebu cattle and introgression of the taurine from the Near-East [7]. As a result, Ethiopia harbors diversified cattle populations [11]. There are 33 morphologically recognized cattle populations in the country (http://dad.fao.org/: accessed January 04, 2017) which are classified into humpless Shorthorn, zebu (large East-African zebu, small East-African zebu), zenga and sanga types [12]. Both the mitochondrial *d*-loop region and nuclear genomic information concurred that there has been extensive hybridization among the indigenous cattle populations in Ethiopia and led to have high level of admixture [6,11,13,14].

Cytochrome *b* (*cyt* b) and *d*-loop regions of mtDNA play significant role in unraveling the population history of livestock species. Using the *d*-loop region of mtDNA, history of genetic diversity and maternal origin of 10 Ethiopian indigenous cattle populations have been reported [11]. However, there is no any report conducted on Ethiopian indigenous cattle populations yet using *cyt* b region to further uncover the maternal origin and population dynamics despite the fact that Ethiopia in particular and the East African region as whole are considered the hybridization zone for both *Bos-taurus* and *Bos indicus* cattle groups [6]. Therefore, in the current study, we sequenced the mitochondrial *cyt* b region aiming to further unveil the genetic diversity, phyogenetic relationship, maternal origin and population expansion of the indigenous cattle in Ethiopia. It also complements information to the findings reported on the diversity of the cattle populations using the *d*-loop and autosomal DNA information [6,11,13,14]. Moreover, there is limited information on the cytochrome region of cattle mtDNA at global level hence this work could provide additional insight about the historical dynamics of the cattle in Ethiopia and in the Horn of Africa at large.

## MATERIALS AND METHODS

Blood samples were collected from 76 animals of Abigar, Boran, Guraghe, Horro, Sheko and one cross bred cattle populations. The cross bred population was *F*_1_ cross between Holstein Friesian (HF) and Barka (Begait synonymously). The cattle populations included in the study are representative of each cattle group previously described by Rege [12] which include: Boran (Large East African zebu), Guraghe (Small East African zebu), Abigar (Sanga), Horro (Zenga), Sheko (African humpless Shorthorn).

Genomic DNA was extracted using Promega Genomic DNA extraction kit [15]. A complete *cyt b* gene (1,140 bp) was amplified using forward primer: 5′-CCATAAATAGGTGAA GGTTTGG-3′ and reverse primer: 5′-TTGATGGTGAGAC TGCAGTT-3′ [16]. The amplification was performed with the polymerase chain reaction (PCR) premix reaction volume of 20 μL that includes 10 μL ExTaq, 1 μL forward primer, 1 μL reverse primer, 1 μL template and 7 μL of RNA free H_2_O. The PCR cycling profile involved an initial denaturation step at 95°C for 2 min, followed by the first stage of amplification of 30 cycles involving a denaturation step at 94°C for 40 s, annealing at 56°C for 40 s, and extension at 72°C for 90 s. The PCR reaction was completed with a final extension step at 72°C for eight min. The PCR products were analyzed by 1% agarose gel electrophoresis and visualized by ultraviolet illumination after staining with gel rednucleic acid gel stain (Biotium, Hayward, CA, USA). The PCR products with good quality were sent to Sangon Biotech in China (http://www.sangon.com) for sequencing.

The sequences were aligned with CodonCode aligner software (version 6.0.2; CodonCode Corporation, Centerville, MA, USA). The nucleotide and haplotype diversities were generated using DnaSP 10.01 [17] and the population differentiation was evaluated using nested analysis of molecular variance (AMOVA) using Arlequin ver. 3.5.1.2 [18]. Pairwise (*F*_ST_) and Reynold’s distances, neutrality tests and populations expansion events were evaluated using ARLEQUIN. We constructed a neighbor joining (NJ) tree based on the Kimura two-parameter model algorithm in MEGA6 [19] to demonstrate the relationships among the haplotypes found in Ethiopian cattle. Median-joining network was constructed using NETWORK 4.5 software [20] to further clarify the maternal genetic ancestry. Thirty three haplotypes/sequences of *Bos taurus* (GenBank Accession Nos.: AY885283–AY885291, DQ124413, V00654, AF490528, AY676860, AY676861, AY676866, AY526085, DQ124371, DQ124389, DQ124372, DQ124374, AB074962 and AB074967) and *Bos indicus* (GenBank Accession Nos.: NC-005971, AF419237, AF492350, AF531473, AY126697, AY689190, EU096517, EU096518, EU096519) from England, the Netherlands, USA, South Korea, Japan, and China were included in the phylogenetic network analysis for comparison. The reliability of the tree topologies was assessed by 1,000 bootstrap replications.

In addition, we examined the star-like clustering patterns of the population expansion by mismatch distribution analysis in ARLEQUIN. With the same package, the history of the indigenous cattle was inferred using Fu’s *F*_S_ and Tajima’s *D* neutrality tests which were implemented in 1,000 simulations.

## RESULTS

### mtDNA sequence variation and genetic diversity

The full length of the mitochondrial *cyt b* gene, 1,140 bp, was sequenced for 76 animals representing six cattle populations in Ethiopia. Eighteen (18) segregating/polymorphic sites were detected in the entire cyt *b* region. The transition to transversion ratio of polymorphic sites is 5:1 and the average codon bias index was 0.566. From the polymorphic sites, seven of them are parsimony informative sites and the remaining 11 are two variant singleton variable sites.

**Figure 1:**
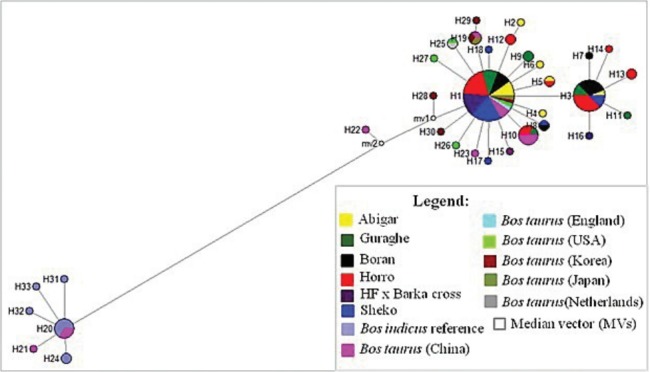
Phylogenetic network graph of Ethiopian cattle populations. The graph was constructed to obtain insights on the genetic relationships between the haplotypes and determine the origin of the maternal lineages in the context of *Bos taurus* and Bos indicus reference haplotypes retrieved from the Gene bank. All the mutations and character states were weighted equally. All haplotypes detected in Ethiopian cattle are clustered with the *Bos taurus* reference haplotypes indicating Ethiopian cattle populations are from *Bos taurus* origin.

From the 18 segregating sites, we observed 18 haplotypes (Table 1), and only five of the haplotypes (H1, H3, H5, H8, and H10) are shared by more than one population. The first haplotype (H1) is the most frequent haplotype observed in 37 sequences and the third haplotype (H3) is the second frequently detected in 16 sequences. Both H1 and H3 are shared by animals from the five indigenous cattle populations. The number of haplotypes in each population ranged from four (Boran and HF×Barka cross) to seven (Horro). Among the thirteen unique haplotypes, H15 and H16 belong to the cross bred population (Figure 1). No median vector is observed among the cattle populations studied indicating absence of unsampled or extinct haplotypes. The average haplotype and nucleotide diversities were 0.7540±0.043 and 0.0010±0.000, respectively (Table 1). The highest and lowest haplotype diversities were observed in Abigar (*H*_d_ = 0.8333±0.127) and Sheko (*H*_d_ = 0.6282±0.143) cattle, respectively.

### Population differentiation and phylogenetic analysis

**Figure 2:**
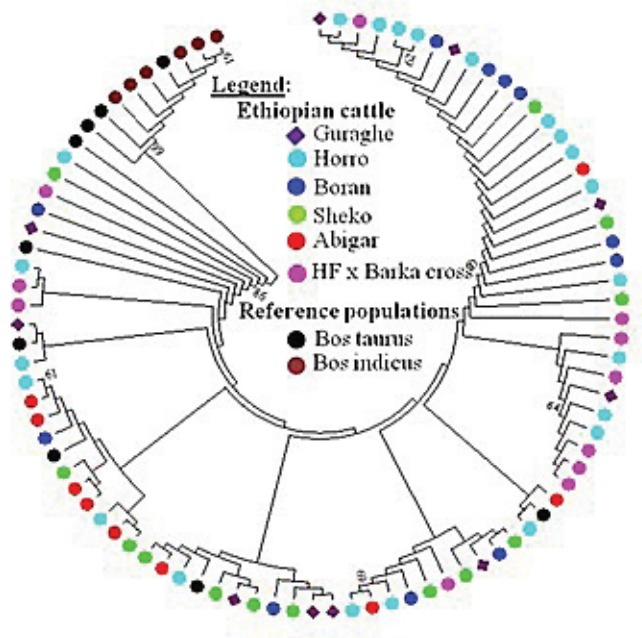
Neighbor joining (NJ) tree of Ethiopian cattle populations. The NJ tree was constructed based on the Kimura two-parameter model algorithm in MEGA6 [19] to demonstrate the relationships among the haplotypes found in the study cattle populations. The NJ tree shows absence of clear phylogeographic structure among Ethiopian cattle populations. Five haplotypes of the *Bos taurus* reference individuals clustered together with Ethiopian cattle; however, no haplotype from the reference *Bos indicus* animals clustered together with Ethiopian cattle.

The AMOVA revealed that there is only 4.55% of variation among the cattle populations studied, and the remaining 94.45% explains variation within the population (Table 2). The overall average pair-wise and Reynold’s distance estimates were 0.0496 and 0.0540, respectively (Table 3). The pair-wise distance estimation indicated that Boran showed highest deviation from Abigar (*F*_ST_ = 0.12446), Sheko (*F*_ST_ = 0.11360), and *F*_1_ (*F*_ST_ = 0.19437). Similar trends were observed using the Reynold’s distance. However, we observed little or no differentiation between Sheko and Abigar, and Boran and Horro cattle populations with both distance measures. On the other hand, both the phylogenetic network and NJ tree analysis show absence of clear phylogeographic structure among the cattle populations (Figures 1, 2). Interestingly, five individuals of the reference *Bos taurus* population clustered together with Ethiopian cattle populations, however, no Ethiopian cattle haplotype matched to *Bos indicus* reference population (Figure 1).

### Population and historical demographic dynamics

**Figure 3:**
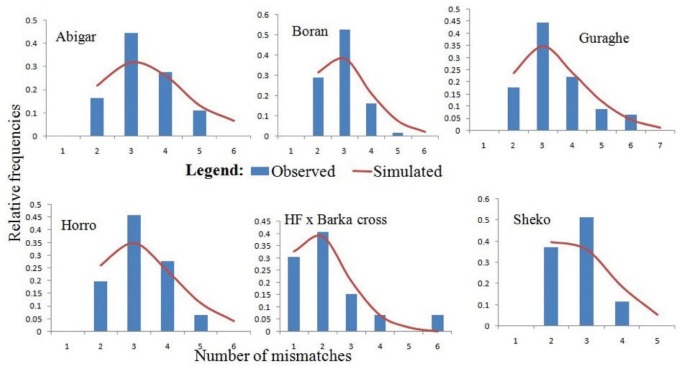
Demographic dynamics among the indigenous cattle populations in Ethiopia. The demographic dynamics of each population was inferred from mismatch distribution patterns following 1,000 coalescent simulations. The figure shows a uni-modal peak indicating a onetime population expansion in each population.

In this study, the uni-modal peak (Figure 3) indicates a single population expansion event held among the cattle populations. There was no significant variation on the overall neutrality and mismatch distribution tests (Tajima’s *D* = −1.0312±0.484 [p>0.05]; *F*_S_ = −1.9374±1.043 [p>0.05]) (Table 4). Similar observation was reported for Chinese cattle [21]. However, there were negative and significant values of Tajima’s *D* test for Abigar and Fu’s *F*_S_ test for Abigar, Horro, and Sheko cattle populations. This indicates natural selection pressure in the populations resulted from excess of rare alleles [22].

## DISCUSSION

### Mitochondrial DNA sequence variation and genetic diversity

In this study, we analyzed the complete mitochondrial *cyt b* gene in five indigenous and one cross bred cattle populations in Ethiopian together with published sequences of *Bos taurus* and *Bos indicus* cattle. The number of segregating sites (*S* = 18) obtained in this study is much lower than those reported for Chinese cattle in the same mtDNA region (*S* = 105) [21], but higher than the segregating sites (*S* = 3 from 18 animals) reported for Leiqiong cattle (*Bos indicus* type) of China [16]. The transition to transversion ratio (5:1) was slightly lower than the value obtained for Chinese cattle populations (5.8:1) [21]. No transversion mutation was observed on the *d*-loop region of mtDNA in Ethiopian cattle populations [11]. Whereas, 62 polymorphic sites (52 transitions, five transversions, five indels) were detected in the *d*-loop analysis of Kenana and Butan cattle populations in the neighboring Sudan [23]. In the current study, we observed 18 haplotypes (H = 18) which is lower than the haplotypes reported for Chinese cattle (H = 47) in same target region [21], however, higher than the haplotypes (H = 3) reported for Leiqiong cattle [16]. In 117 *d*-loop sequences, 81 haplotypes were identified in 10 Ethiopian indigenous cattle populations [11]. The relative higher number of haplotypes observed in the *d*-loop could be because of high mutation rate of the *d*-loop region compared to *cyt b* [24]. For north Ethiopian cattle populations, 11 indicine and one taurine haplotypes were detected from five Y-chromosome microsatellites analyzed.

The current study revealed that the average haplotype diversity (*H*_d_ = 0.7540) as well as nucleotide diversity (π = 0.00100) (Table 1) are slightly lower than the diversities observed for Chinese cattle (*H*_d_ = 0.848; π = 0.00923) [22]. This could be because of presence of wider gene pool of maternal origins in Chinese cattle compared to Ethiopian cattle. However, lowest average values of haplotype (*H*_d_ = 0.0741) and nucleotide (π = 0.0012) diversities were reported for Leiqiong cattle [16]. This could be because of sampling bias, in which most of the individuals included in the study (n = 18) in the latter cattle population could be collected from similar haplotype origin and as a consequence very few number of haplotypes (H = 3) detected in the *cyt* b region. Based on the analysis of Y-chromosome microsatellites of north Ethiopian cattle populations, the average haplotype diversity was 0.617167±0.02617 [7].

### Population differentiation

The population differentiation analysis revealed very weak populations structure (4.55%) among the cattle populations studied and the highest variation (95.45%) is explained by within population variation (Table 2). However, no (zero) percentage of variation was reported among Ethiopia cattle populations using the *d*-loop [11]. With same target region (*d*-loop), only 2.4% of variation was observed between Kenana and Butan cattle in Sudan [23]. Very low percentage of variation was reported for North Ethiopian cattle based on the analysis of Y-chromosome simple sequence repeats (SSR) markers [7]. Very low population differentiation estimates were also reported based on the nuclear DNA analyses in Ethiopian indigenous cattle populations (SSR markers: 1.3% [13] and 1.1% [25]; single nucleotide polymorphism CHIP panel: 2% [6]). The very weak phylogeographic structure and low genetic differentiation could be a result of a recent common ancestral origin, multiple introgressions and strong genetic exchange among the indigenous cattle populations [6,13,25]. On the other hand, the highest within-individual genetic variability observed in Ethiopian cattle provides an untapped opportunity for adaptation to changing environments and for implementation of within-breed genetic improvement schemes [6] in which genetic variability enables adaptation of natural populations to changing environments. Overall, the relationships among Ethiopian cattle populations, which represent a mosaic of the humped zebu and taurine, reflect their evolutionary history of origin and admixture rather than their phenotype based categorization as zebu, sanga and zenga [6], as we concluded in this study.

Among Ethiopian indigenous cattle populations, the pair-wise (*F*_ST_) and Reynold’s distances vary from −0.01113 (between Sheko and Abigar) to 0.12446 (between Abigar and Boran) and zero (between Boran and Horro, Abigar, and Sheko) to 0.13292 (between Boran and Abigar), respectively (Table 3). However, we observed no differentiation between Sheko and Abigar despite the fact that Abigar cattle have the highest indicine allele frequencies compared to other sanga cattle in Ethiopia [26]. This could be due to two reasons: i) Sheko is an African short horn taurine and Abigar, which is a sanga type, is produced from introgression of *Bos taurus* and zebu (*Bos indicus*) groups [26]; and hence, Abigar partly shares same ancestral origin of Sheko; ii) it could be because of high gene flow of Abigar cattle population towards Sheko breeding tract. A recent extensive genome-wide study revealed that there is very high allelic gene flow between Sheko and other local cattle populations as a consequence Sheko cattle population is getting assimilated [6]. The close geographical proximity between the two cattle populations (Sheko and Abigar) could facilitate the gene flow easily since isolation-by-distance plays fundamental role for differentiation of populations.

On the other side, we observed highest differentiation between Boran and Abigar (*F*_ST_ = 0.12446), and Boran and Sheko (*F*_ST_ = 0.1136). This could be because of low gene flow, geographical isolation, ecological factors and morphological adaptation to local conditions. The Boran cattle, a humped breed reflecting indicine introgression, carry genes adaptive to harsh environment and heat tolerance developed by a long-term natural selection, display high resistance to heat and ticks and good productivity on poor forage and low amounts of water, whereas, Sheko and Abigar are found in humid and forest areas where burden of tsetse flies is high [27].

### Phylogenetic analysis

We constructed median joining network to reveal the phylogenetic relationships among the cattle populations studied (Figure 1). The network illustrates a star-like pattern indicating a population expansion event and this strengthened by uni-modal population expansion event detected (Figure 3). This observation is consistent with the *d*-loop region of the mtDNA reported on Ethiopian indigenous cattle populations [11]. In addition, among the five globally identified maternal origins (T, T_1_, T_2_, T_3_, and T_4_) of *Bos taurus* and two lineages (I_1_ and I_2_) of *Bos indicus* [2,28,29], all the 81 haplotypes detected in ten Ethiopian cattle populations clustered with Haplogroup T1 [11]. However, double peaks were reported for North Ethiopia cattle populations using Y chromosome microsatellite haplotype mismatch distribution analysis [7]. All the detected haplotypes (H = 50) in Kenana and Butan cattle of Sudan belong to haplogroups T1 [24]. In the *d*-loop analysis of Ethiopian cattle, a major haplotype occurred at the H1 with the highest frequency which showed a broad geographic distribution and represents the possible ancestral haplotype of T1 lineage [11]. In the current study, we also observed the highest frequency at the H1 and the second highest haplotype occurrence at the H3 (Figure 1).

Interestingly, in the current study, nine of haplotypes of the *Bos taurus* reference sequences clustered with Ethiopian indigenous cattle populations. All these haplotypes are from USA, European and Asian *Bos-taurus* cattle. In contrast, we did not observe any haplotype from *Bos indicus* reference sequences being shared or clustered with Ethiopian indigenous cattle despite the fact that four of the indigenous cattle populations included in this study are from *Bos indicus* group. Despite the proportion of variation of influence, influence of European taurine on North Ethiopian cattle was reported but exhibited a weaker influence compared to Asian taurine origin [28]. According to Bradley et al [30], African *Bos taurus* and African *Bos indicus* share the same African set of taurine mitochondrial DNA haplotypes suggesting the pattern of zebu influence on the African continent was a process of introgression rather than replacement of African taurine cattle with unmixed Asian zebu as we also observed in this study. In line with this, the genome-wide analysis unveiled presence of substantial taurine introgression in Ethiopia zebu, sanga, and zenga cattle [6]. As a result of subsequent introgression between the African taurine type and South-Asian zebu (*Bos indicus*) the existing populations, for instance Ethiopian cattle, have been created with different proportions of taurine and indicine backgrounds [1,9]. The north Ethiopian cattle breeds have been heavily (>90%) influenced by Zebu, followed by African, European and the Near-Eastern tourine, respectively [25]. A recent worldwide admixture and divergence analysis uncovered that the indicine ancestry in African cattle is higher in East Africa (74%) [10], which confirms the former premise that explains the East African region could be considered as the cradle of African zebu [3]. However, Edea et al [6] still argues that the stronger influence of zebu ancestry in Ethiopian cattle can be attributed to the replacement of taurine by *Bos indicus* than introgression due to adaptation of zebu to harsh environments like tolerance for heat, ticks, drought and poor forage.

Over all, the cluster of the haplotypes detected in Ethiopian indigenous and the cross bred cattle populations to the *Bos taurus* origin of the reference populations asserts the former finding that shows all African cattle possess taurine humpless longhorns (*Bos taurus*) [11,26]. In line with this, no haplotype which clustered to *Bos indicus* type was observed from 10 indigenous cattle populations analyzed using the *d*-loop region and attributed to the challenge of recurrent drought and rinderpest epidemics [11]. This could lead to mtDNA more sensitive to demographic expansion like population fragmentation and bottleneck, the east African region suffer [11]. In contrast, the very limited number of taurine alleles of the Y-chromosome reported for the north Ethiopian cattle population could be a result of recent crossbreeding or incomplete introgression of zebu patrilines [7]. Overall, the different data sets reported on Ethiopia cattle populations indicate the distribution of taurus cattle vary in the different parts of the country.

### Historical dynamics of the cattle populations

The bell-shaped curve of the population dynamics obtained from the mismatch distribution analysis reveals presence of population expansion (Figure 3). However, the p-values of the sum of square deviation (*SSD* = 0.01901±0.009; p = 0.375) and raggedness index (*r* = 0.1564±0.039; p = 0.318) tests were not significant. On the other hand, negative values for all the populations studied and negative and significant values of the coalescent-based neutrality tests (Tajima’s *D* for Abigar, *F*_S_ value for Abigar, Horro, and Sheko) obtained also suggest existence of a population expansion event.

The absence or little deviation of Horro from Boran could mean that Horro’s zebu line could be Boran. Horro is from zenga group: cross of zebu and sanga cattle [12]. We exhibited similar observation between Sheko and *F*_1_ populations which could be because of their *taurus* background. On the other hand, moderate population differentiation was observed between tsetse susceptible *Bos indicus* representative (Boran) and resistant *Bos taurus* group (Sheko) and *F*_1_ populations (Table 3). This observation is strengthened by highest (0.229) Nei’s corrected distance observed between Boran and Sheko using 10 microsatellite markers [14]. Solomon [14], reported very narrow among population variation (2.2%) for the same study populations (Boran, Abigar, Guraghe, Horro, and Sheko) with the same SSR markers.

In conclusion this study revealed highest mtDNA variations among Ethiopian cattle populations and the haplotypes clustered into a haplogroup which goes in line with previous reports on East African cattle populations [11,24]. Moreover, our finding asserted that the cattle populations studied are from the *Bos taurus* ancestral origin given the fact that they are highly influenced by the *Bos indicus* group. The dynamics of population history showed presence of a onetime but rapid and recent population expansion in all the cattle populations studied.

## Figures and Tables

**Table 1:** Genetic diversity indices of Ethiopian cattle populations using cyt b region of mtDNA

Morphological group	Population	N	S	H	K	Hd	π
Sanga	Abigar	9	6	6	1.333	0.8333±0.127	0.0012±0.0003
Large East African zebu	Boran	11	3	4	0.910	0.709 1±0.099	0.0008±0.0002
Small East African Zebu	Guraghe	10	5	5	1.422	0.8222±0.097	0.0013±0.0003
Zenga	Horro	22	6	7	1.208	0.8009±0.059	0.0012±0.0002
Cross bred (*F*_1_)	HF×Barka	11	4	4	0.982	0.6727±0.123	0.0009±0.0003
African humpless Shorthorn	Sheko	13	4	5	0.744	0.6282±0.143	0.0007±0.0002
	Overall	76	18	-	1.138	0.7540±0.043	0.0010±0.0001

*Cyt*, cytochrome; N, number of samples; S, segregating sites; H, number of haplotypes; K, nucleotide differences; *H*_d_, haplotype diversity; π, nucleotide diversity; *F*_1_, Holstein Friesian (HF)×Barka cross.

**Table 2:** Analysis of molecular variance (AMOVA) of Ethiopian cattle populations

Source of variation	Sum of square	Variance components	Percentage of variation
Among populations	4.353	0.02612	4.55206
Within populations	38.331	0.54759	95.44794
Total	42.684	0.5737	-

**Table 3:** Pairwise (*F*_ST_) (below diagonal) and Reynold’s (above diagonal) distances of Ethiopian cattle populations

Population	Abigar	Boran	Guraghe	Horro	*F*_1_	Sheko
Abigar	-	0.13292	0.01560	0.04091	0.03292	0.00000
Boran	0.12446	-	0.03144	0.00000	0.21613	0.12059
Guraghe	0.01548	0.03095	-	0.00010	0.03016	0.01688
Horro	0.04009	−0.01067	0.00010	-	0.07966	0.04540
F_1_	0.03239	0.19437	0.02971	0.07657	-	0.04716
Sheko	−0.01113	0.11360	0.01673	0.04438	0.04606	-

F1, Holstein Friesian×Barka cross bred.

**Table 4:** Mismatch distribution and neutrality tests of Ethiopian cattle populations

Populations\models	Tajima’s *D* (p-value)	*F*_S_ (p-value)	Raggedness index (p-value)	Sum of square deviation (p-value)
Abigar	−1.7278 (0.017)	−3.3289 (0.002)	0.1451 (0.460)	0.0162 (0.470)
Boran	−0.3848 (0.341)	−0.9398 (0.116)	0.2096 (0.200)	0.0321 (0.190)
Guraghe	−0.7832 (0.241)	−1.3926 (0.105)	0.1432 (0.350)	0.0161 (0.450)
Horro	−0.8250 (0.232)	−2.6506 (0.021)	0.1497 (0.090)	0.0187 (0.140)
HF×Barka cross	−1.0292 (0.206)	−0.7773 (0.150)	0.0998 (0.690)	0.0061 (0.750)
Sheko	−1.4371 (0.095)	−2.5349 (0.002)	0.1912 (0.120)	0.0248 (0.250)
Mean±SD	−1.0312±0.484 (0.189)	−1.9374±1.0431 (0.066)	0.1564±0.039 (0.318)	0.01901±0.009 (0.375)
